# The experiences and beliefs of older people in Scottish very sheltered housing about using multi-compartment compliance aids

**DOI:** 10.1007/s11096-017-0580-x

**Published:** 2018-01-13

**Authors:** Derek Stewart, Kathrine Gibson Smith, Joan MacLeod, Alison Strath, Vibhu Paudyal, Katrina Forbes-McKay, Scott Cunningham, Katie MacLure

**Affiliations:** 1 Robert Gordon University, Aberdeen, Scotland, UK; 2 University of Birmingham, Birmingham, England, UK

**Keywords:** Ageing, Behavioural medicine, Geriatrics, Patient adherence, Patient education, Primary care, Scotland

## Abstract

*Background* Multi-compartment compliance aids (MCAs) are promoted as a potential solution to medicines non-adherence despite the absence of high quality evidence of effectiveness of MCA use impacting medicines adherence or any clinical outcomes. Furthermore, there is a lack of qualitative research which focuses on the perspectives of older people receiving MCAs. *Objectives* To describe experiences and beliefs surrounding very sheltered housing (VSH) residents’ use of MCAs with emphasis on issues of personalisation, reablement, shared decision-making, independence and support. *Setting* VSH in north east Scotland. *Methods* Qualitative, face-to-face interviews with 20 residents (≥ 65 years, using MCA > 6 months) in three VSH complexes. Interviews focused on: when and why the MCA was first introduced; who was involved in making that decision; how the MCA was used; perceptions of benefit; and any difficulties encountered. Interviews were audiorecorded, transcribed and analysed using a framework approach. *Main outcome measure* Experiences and beliefs surrounding use of MCAs. *Results* Nine themes were identified: shared decision-making; independence; knowledge and awareness of why MCA had been commenced; support in medicines taking; knowledge and awareness of medicines; competent and capable to manage medicines; social aspects of carers supporting MCA use; benefits of MCAs; and drawbacks. *Conclusion* Experiences and beliefs are diverse and highly individual, with themes identified aligning to key strategies and policies of the Scottish Government, and other developed countries around the world, specifically personalisation shared decision making, independence, reablement and support.

## Impacts on practice

- Given that the experiences and beliefs surrounding the use of MCAs by elderly are diverse and highly individual, focus should be placed on a person centred approach to care.
- Health and social care professionals involved in aspects of the supply of MCAs should consider issues of personalisation, shared decision-making, independence, reablement and support.
- Issues like peronalisation, independence and reablement relate to autonomy, competence, and relatedness which are central to Self Determination Theory. Hence this theory may provide a framework to support appropriate use of MCAs.

## Introduction

The World Health Organization (WHO) *World Report on Ageing and Health* highlights that globally, the number and proportion of older people is increasing markedly [1]. In Scotland, the number of people 65 years and over is estimated to increase by 53% between 2014 and 2039 [2]. In 2009, the Scottish Government Ministerial Strategic Group for Health and Wellbeing developed a strategy, *Reshaping Care for Older People*, with the goal to ‘optimise the independence and wellbeing of older people at home or in a homely setting’ [3]. The strategy places much emphasis on a health and social care shift towards ‘personalisation’, whereby people become more involved in how services are designed and receive the support that is most suited to them. More recently, the focus for the support of older people has been based on ‘reablement’, to assist older people to learn or relearn those skills needed for a successful and fulfilling life [4].

Personalisation, reablement, shared decision-making, independence and support may be viewed in the context of Self Determination Theory (SDT), a theory of motivation and human behaviour [5]. SDT outlines three key constructs which are considered intrinsic psychological needs and which contribute to enhanced wellbeing: competence, autonomy, and relatedness. Autonomy refers to a need to feel volitional and responsible for the execution of a behaviour; competence concerns the need for individuals to feel effective in interactions with their environment; and relatedness, the need to feel a sense of belongingness and connectedness with others. The theory further highlights the importance of the role of the social environment in fostering the three psychological needs [6, 7].

These principles of personalisation, reablement, shared decision-making, independence and support are of direct relevance to the use of medicines in older people [8, 9], particularly given the extent of medicines use in this population. Multimorbidity, defined as ‘the co-occurrence of two or more chronic medical conditions in one person’, is highly prevalent in older people, increasing rapidly with age in terms of both prevalence and the number of morbidities [10]. Increases in multimorbidity in older people are mirrored by the increasing numbers of prescribed medicines, as evidenced from data published in the United Kingdom (UK) [11, 12], United States [13], and Europe [14].

Medicines non-adherence is one potential consequence of the increasing medicines burden in multimorbid older people [15, 16], with data suggesting that between 50 and 80% of those with chronic conditions may be non-adherent [17]. A number of interventions have been proposed as potential solutions to non-adherence in older people [18, 19]. Multi-compartment compliance aids (MCAs), also referred to as Monitored Dosage Systems and Dose Administration Aids, are repackaging systems for solid dosage form medicines, such as tablets and capsules, where the medicines are removed from manufacturer’s original packaging and repackaged into the MCA [20]. While these are promoted as a potential solution to non-adherence, the Royal Pharmaceutical Society has expressed concern that pharmacy supplied MCAs have ‘become regarded as a panacea for medicines use and often integrated into practice and service policy without giving due consideration to the alternatives’ [20].

Systematic reviews published in 2011 and in 2016 highlighted the lack of evidence of effectiveness of MCA use impacting medicines adherence or any of the clinical outcomes studied [21, 22]. In addition, MCA use in older people may be linked to reduced medicines related knowledge, thought to be due to not recognising the different medicines within the MCA [23]. To date there is a dearth of research which has focused on the perspectives of older people receiving MCAs. One study of older people living independently and an unrelated sample of health professionals involved in MCA provision identified mixed views on whether MCAs helped or hindered in maintaining independence and control over medicines [24].

Given the predictions of increased numbers of older people globally, the extent of medicines use, the potential for non-adherence and consequent MCA use, there is a need to ensure that older people are involved in any decision to commence an MCA. Ideally that decision should also be centred on the principles of personalisation, reablement, shared decision-making and independence.

We recently reported a case study of older people provided with MCAs, their carers and health professionals, the aim of which was to explore the factors influencing MCA use [25]. Goals of use related to promoting adherence and safety, with less emphasis on independence. Beliefs of consequences related to these goals were considered of value, with additional consequences of concern around reduced awareness of medicines and complexities of changing medicines. In this paper, we focus on the perspectives of the older people studied.

## Aim of the study

The aim was to describe the experiences and beliefs of older people surrounding the use of MCAs, with emphasis on issues of personalisation, reablement, shared decision-making, independence and support.

## Ethics approval

This study was approved by the National Health Service (NHS) North of Scotland Research Ethical Review Service (14/NW/1168) and NHS Grampian Research and Development Committee (2014RG002).

## Method

### Design

This was a qualitative study comprising individual face-to-face interviews conducted by a researcher with extensive experience in qualitative interviewing.

### Setting

The study was conducted within ‘very sheltered housing’ (VSH) complexes in the north east of Scotland. In the UK, ‘sheltered housing’ describes rented housing for older and/or disabled or other vulnerable people, usually in grouped developments. VSH generally has all the features of sheltered housing, but has enhanced care and support through the service of extra wardens, full-time carers, assistance with everyday living, including assistance with medication, and provision of meals.

### Inclusion and exclusion criteria

Residents of three VSH complexes were included if they were aged 65 years or over and had been using an MCA for 6 months or more. Potential participants were identified by the VSH senior carer, who excluded those known to have significant cognitive or welfare issues.

### Recruitment

The National Health Service employed primary care lead pharmacist for the area (known to the VSH senior carer) met each screened resident, inviting participation. If the resident was in agreement, signed, informed consent was obtained and a convenient date, time and location for the interview arranged.

### Interview schedule

Questions in the semi-structured interview schedule focused on: when and why the MCA was first introduced; who was involved in making that decision; how the MCA was used; perceptions of benefit; and any difficulties encountered. The interview schedule was reviewed independently, for credibility, by four individuals with expertise in health services research. This was followed by piloting with two participants fulfilling the eligibility criteria who were then excluded from the main study.

### Data generation

Interviews of approximately 30 min duration took place during September to November 2014. Each interview was audio-recorded, with permission, transcribed verbatim and checked for transcribing accuracy by a member of the research team. Recruitment and data generation continued to the point of saturation when no further themes were emerging from three consecutive interviews.

### Data analysis

Members of the research team met to agree consistency of the initial coding framework. Data were analysed using the Framework Approach of: data familiarisation (e.g. reading of transcripts); identifying constructs (e.g. coding relevant statements); indexing and charting (e.g. refining codes and identifying themes and sub-themes); mapping (e.g. refining themes and sub-themes); and interpreting (e.g. interpretation of broader picture of themes and sub-themes). Transcripts were coded independently by two researchers. Whilst SDT was used when interpreting the results, it was not used when planning the study and conducting the interviews.

## Results

### Demographics

Twenty residents across three VSH sites (A, B and C) participated; the majority (n = 15) were female, there were no refusals. It was perceived that data saturation, whereby no new themes emerged, occurred after interviewing twenty residents. All were aged 65 years and over, had been using an MCA for a minimum of 6 months, and the VSH senior carer had screened residents to avoid those known to have cognitive impairment or welfare issues. Table 1 gives the nine key themes and corresponding sub-themes identified relating to residents experiences of and beliefs around using MCAs. There were often contradictory experiences and beliefs within the themes.

**Table 1:** Themes and sub-themes

Theme	Sub-theme	Illustrative quote
Shared decision-making	Involved in decision to start MCA	Interviewer “So when you say ‘we thought’, were you involved in the decision?” Interviewee “Yes” Case 5 at D
	Not involved in decision to start MCA	“No, I don’t know how it came about at all, you know?’’ Case 5 at C
	Cannot recall if involved in decision to start MCA	“Now that’s so long ago, I don’t know” Case 3 at D
Independence	Lack of confidence	“[carers] Canny [cannot] trust me” Case 1 at D
	Loss of independence	“That’s what’s getting me down. I canna [cannot] do the things I used to do” Case 2 at D
	No loss of independence	“No, no [feel as though independence is going]. I’m used to it now” Case 2 at K
	Trust in carer	“I rely on the carers” Case 4 at K
Knowledge and awareness of why MCA commenced	Aware of purpose of MCA	“Well, it’s for, it’s for segregating your medication and letting you know what’s what in it” Case 5 at C
	Unaware of why MCA started	“I think they just said this is how it’s going to be and that’s it” Case 3 at D
Knowledge and awareness of medicines	Awareness of what medicines are prescribed for	“But I mean I know exactly what the tablets are for or how many I need” Case 9 at D
	Awareness of what some medicines are prescribed for	“No, I can differentiate with most of them. The ones I can’t is the ones that are all white” Case 3 at D
	Unaware of what medicines are prescribed for	“Furosemide was that ein [one]. Da ken [I don’t know] what it’s for” Case 4 at K
	Cannot recognise all medicines	“No, no I dinna ken [don’t know] them all’’ Case 2 at D
	Recognises medicines	“Well there was one occasion when they were giving me tablets and I says ‘oh there’s something missing here, I’m short’, and it was, these white paracetamol” Case 5 at C
	Awareness of when to take medicines	“Well, actually I’m awful good at phoning. So if she’s [carer] not here by half past 5 she’s kens [knows] there’ll be a ‘phone call” Case 3 at C
Support in medicines taking	Involvement with taking medicines	“That’s your responsibility, we’ve given you it, if you don’t want to take it just now and you’re going to take it later that’s your responsibility, you can say yes or no if you want to take it and I says well” Case 1 at K
	Reduced involvement with taking medicines	“No, I never touch them [medicines]” Case 4 at K
Competent and capable to manage medicines	Feels capable/confident	“I’m lucky I haven’t got anything wrong with my mind, I’m spot on and of course it’s like everything else, I’m fine for my memory” Case 3 at C
	Forgets medicines	“But sometimes now I think I’m glad I gave them over because sometimes I think I might forget them” Case 5 at D
	Illness/dexterity	“I canna [cannot] see, that’s why I need” Case 9 at D
	Managing meds by self	“My inhalers, I order them myself” Case 5 at D
	Issues with medicines prior to MCA	“Well, when you’re taking them out of a bottle, you know, you let it fall and sometimes you canna [cannot] find, it” Case 1 at C
Social aspects of carers supporting MCA use	Social aspect of MCA	“Aye [yes], but when [name] came up and said ‘I think it [having the MCA] would be better’. So I said ‘fair enough’ because when they start that it means a girly [carer] to come up” Case 6 at D
Benefits of MCAs	Ease of using MCA	“Well we found them easier to open, they’re all together in the one bit and you open them” Case 2 at K
	Safety of MCA	“Well for one thing the medical bottles, as you know, very similar and when you’re in a hurry, which most of them are, it’s an accident waiting to happen” Case 1 at K
	MCA alleviates worry about taking medicines	“The one good thing about it is the blister pack starts on a Monday but they’re delivered on a Wednesday. Now, to me that’s a good thing because I’m always worried come Saturday or Sunday, where’s my tablets” Case 4 at D
	MCA promotes control over medicines	“Well, it’s for my medicine, my tablets and I think it’s a lot better than bottles and that, cause you’re not losing them” Case 4 at C
	MCA increases confidence	Interviewee: “It tells you you’re supposed to take…you canna [cannot] miss any [medicines], they’re all there…” Interviewee: “It gives you confidence” Interviewee: “Aye, you ken [know] it’s [the medicine] there” Case 4 at K
Drawbacks of MCAs	Issues with using MCA	“I’ve lost a few little ones, dropped on the floor, like that thyroid tablets, their tiny” Case 4 at K
	Complexities of MCA	“…and paracetamol, they’re not in the blister pack, they’re just loose and they give them as well” Case 2 at D

### Shared decision-making

Although some residents felt that they had been involved in the decision to start an MCA, most reported that they could not recall if they had been involved, or that they had limited or no involvement in the decision,

No, I don’t know how it came about at all, you know? (Case 5 at C).

One resident highlighted the involvement of others in the decision,

My brother was speaking about it and he says ‘you’d be better getting that’. And I says ‘I’ll leave it, one of the lassies’ll [carers will] tell me’ and it was (name), it was (name) that said that I’d be better. (Case 6 at C)

### Independence

Some described a perceived loss of independence as a consequence of not being involved in the administration of their medicines, with one expressing frustration over the lack of control. Others, however, noted that they did not feel that they had lost any independence by not being in control of their medicines. One was of the view that having the MCA had promoted independence,

I prefer the blister pack, because I mean your dosage is done, it’s taken care of and from time to time, if you want or if you feel capable, you can open it and you look and you see, oh yes, that’s right. (Case 1 at A)

### Knowledge and awareness of why MCA commenced

A number of residents recounted their understanding of the purpose of the MCA, that it was required due to reduced capabilities attributable to loss of dexterity, illness and issues with remembering to take medicines. Whilst some residents were aware of why and when they had been provided with an MCA, others were unable to recall when and why it had been started,

Not really. I suppose in case you forgot [medicines]. I don’t know, to take them. (Case 1 at B)

### Knowledge and awareness of medicines

Residents varied in their knowledge and awareness of their medicines, with some having high levels of awareness, knowing what medicines were prescribed for and how much to take. Others, however, reported that with the MCA they had difficulty recognising their medicines,

No, no, I just, well I don’t know if I get anything for it, there’s a lot of tablets I don’t know what they’re for. (Case 2 at C)

### Support in medicines taking

There was diversity over the extent to which residents were actively or passively involved in medicines taking. Some discussed that they delayed taking their medicines or chose to take them at certain times, demonstrating ownership and empowerment. For most, their carers prompted or supported medicines taking from the MCA,

They, they do everything, I don’t have a blister pack in my hand at all. (Case 7 at C)

### Competent and capable to manage medicines

Several residents reported that they were confident that they were competent to manage their medicines. Others reported that they were responsible for taking and managing medicines that were not stored in the MCA. As one resident stated,

…because they don’t realise, I mean I’m 79 year old now, I know what I’m doing, it’s life, I’ve still got my brain up here (Case 6 at B)

### Social aspects of carers supporting MCA use

The social aspects of having carers support medicines administration from MCAs were highlighted where the benefit of having company was described,

But it’s as much because you get the company of somebody coming in four times a day. It’s good for somebody to come. You’re speaking for a wee while. (Case 6 at B)

### Benefits of MCAs

MCAs were perceived positively, with residents describing associated benefits, for both carers and themselves, including increasing medicines adherence, enhanced safety and prevention of lost medicines,

Well they make certain that people who might not have full comprehension doesn’t take their tablets at the wrong time and in the wrong sequence (Case 3 at C)

### Drawbacks of MCAs

Whilst a number of residents reported no disadvantages to MCAs, the complexities of MCAs were highlighted by several residents, who reported that some medicines were not in their MCA. It was also highlighted by some residents that carers often experienced difficulties in opening and using MCAs,

The lassies [carers], the lassies sometimes have a job themselves. (Case 6 at B)

## Discussion

This study has provided an in-depth description of the perspectives of residents of VSH surrounding their experiences and beliefs of using MCAs. Nine key themes were identified: shared decision-making; independence; knowledge and awareness of why the MCA had been commenced; support in medicines taking; knowledge and awareness of medicines; competent and capable to manage medicines; social aspects of carers supporting MCA use; benefits of MCAs; and drawbacks of MCAs. There were many examples of diverse, and often polarised, experiences and beliefs within each theme.

### Interpretation

Personalisation, shared decision-making, independence, reablement and support are key aims of strategies and policies of the Scottish Government [3, 4, 9] and other developed countries around the world [26]. Many of the themes of experiences and beliefs which emerged in this study align to these elements. Shared decision-making and personalisation were apparent in those reporting involvement in the decision to commence an MCA. This was interpreted as an indication that involvement in medicines taking was facilitated through MCA use in those who were competent and capable. Similarly, some residents felt that their independence was promoted through MCA use and many recounted many areas of support derived from MCA use including directly in medicines taking, feelings of empowerment and the carers aiding medicines administration. There were also aspects of reablement, the relearning of skills, in terms of the use of MCAs making medicines taking more manageable particularly on occasions where there were issues with memory, dexterity, eyesight, stress and being overwhelmed. Residents also described their awareness of their medicines and being able to check that the medicines in the MCA were correct, given at the appropriate time and in the prescribed amount. However, as noted earlier, there were examples of polarised experiences and beliefs within the themes with a lack of shared decision-making, personalisation and empowerment, and general feelings that independence was reduced through MCA use. These are important considerations for all health and social care professionals involved in the use of MCAs. These findings are of particular importance for pharmacists and pharmacy staff given their acknowledged roles in the provision and review of MCAs in older people [27]. It may be beneficial for pharmacists and pharmacy staff to explore patient issues around MCA us in an effort to promote shared decision-making.

The issue of non-adherence in those with chronic conditions is widely acknowledged [15]. Given the predictions of the increasingly older population, combined with prevalence of multimorbidity and numbers of prescribed medicines, there is potential for these non-adherence statistics to increase. MCAs represent one potential solution for those whose non-adherence is non-intentional and indeed promoting adherence was noted as a benefit in this study and others [24, 25]. While systematic reviews have failed to demonstrate objectively that MCAs improve adherence [21, 22], there are particular issues around obtaining valid and reliable measures of adherence in those receiving MCAs. The perspectives of the individual and their attendant carers and health professionals should therefore not be underestimated. There is a need to ensure, however, that MCAs are targeted to the correct individuals, and there is strong evidence of the need for review of all medicines [28–31].

It is clear from the findings of this study that the experiences and beliefs around MCA use in older people are very individual and hence diverse. The elements of personalisation, shared decision-making, independence, reablement and support may all relate to, and be interpreted, by SDT. The themes ‘knowledge and awareness of why MCA commenced’, ‘support in medicines taking’ and ‘independence’ may all be encapsulated within the SDT need for autonomy. Specifically, in relation to MCAs, residents demonstrated varying levels of autonomy in relation to management of their medicines with some reporting a high degree of responsibility and others, limited involvement in the process. The themes ‘knowledge and awareness of medicines’, ‘competent and capable to manage medicines’ may pertain to the competence need of SDT. Residents often demonstrated a desire to manage their own medicines since they felt capable of doing so, however, conversely, others were relatively happy to absolve responsibility of managing their medicines since they believed they were less capable. The ‘social aspects of carers supporting MCA use’ may relate to the relatedness need outlined in SDT. Residents discussed the role of the carer in managing medicines and how this was considered to be a more social aspect of having an MCA.

Perhaps, prior to initiation of an MCA, it may prove beneficial for health and social care professionals to consider the individual needs of residents in relation to using MCAs within the context of SDT. Performing tailored theoretical analyses of residents’ psychological needs may aid personalised goal setting and plans for monitoring MCA use thereafter. The tenet of personalisation may be particularly important moving forward. Custer et al. in a study of need fulfilment in nursing home residents using SDT as a foundation, highlighted the individuality and variability in how residents perceived the importance of autonomy and competence. Although being provided with the opportunity to make decisions and complete tasks independently were critical factors in ensuring fulfilment of needs for some, they were not priorities for others [32].

### Strengths and weaknesses

There are several strengths to this study which are based around the measures to promote credibility (e.g. adopting research methods well established in qualitative investigation, expert review of the interview schedule, iterative questioning, encouraging residents to be frank in their responses), dependability (e.g. an experienced qualitative interviewer) and transferability (e.g. detailed description of setting and participants). It is also highly likely that the sample size was sufficient for saturation of themes and issues. There is also a lack of qualitative research in this field hence the findings contribute to this limited evidence base. In particular, the emphasis on the residents’ perspectives of experiences and beliefs complement our overall case study based approach which sought to elucidate behavioural determinants of MCA use [25]. The study adds to existing literature on MCAs in that it has contributed to enhanced understanding of MCA use in older adults. There are, however, study limitations most notably the issue of transferability of the findings to older people resident in other settings and countries with different health and social care systems. Whilst the results were viewed through SDT, the theory did not inform the design of the study and hence, greater depth may have been attained around the three tenets of theory had it been. Furthermore, the findings may be impacted by recall bias, particularly around issues of reasons for commencing MCAs which may have occurred in the distant past.

### Further research

Further research should now focus on exploring SDT within the context of MCA use in older people. Personalisation should be a key consideration since it would assist in ensuring that an individual’s priorities, in terms of psychological needs, correspond with the support that is provided in relation to management of medicines. Hence, wellbeing may be optimised within the population by maintaining congruence with regard to both preferences for managing medicines and experience.

## Conclusion

The experiences and beliefs around MCA use in older people are diverse and highly individual. The themes identified aligned with key strategies and policies of the Scottish Government, specifically personalisation shared decision-making, independence, reablement and support.
